# Microwave Acid Sample Decomposition for Elemental Analysis

**DOI:** 10.6028/jres.093.041

**Published:** 1988-06-01

**Authors:** H. M. Kingston, L. B. Jassie

**Affiliations:** Center for Analytical Chemistry, Inorganic Analytical Research Division, National Bureau of Standards, Gaithersburg, MD 20899

Appropriate sample preparation is essential to achieve both accuracy and precision in the analysis of materials. This preliminary step is one of the most time-consuming parts of many analyses and has become the rate limiting step for such multi-element techniques as ICP, XRF, and ICP-MS. Acid dissolution of biological and botanical samples can take from 4 to 48 hours using classical digestion techniques. Many of these same samples require only 10 to 15 minutes with microwave digestions, dramatically reducing preparation times. Volatile elements such as selenium, phosphorus, tellurium, and vanadium can be retained quantitatively in a sealed vessel using microwave decomposition prior to instrumental analysis [1]. The technique has been tested on all the major sample types including biological, botanical, geological, alloy, and glassy samples and has demonstrated advantages for each of these sample groups.

The development of real-time monitors for temperature and pressure in the microwave environment permits the investigation of closed vessel digestion using microwave energy as the heat source. It is necessary, however, to have an understanding of the fundamental concepts controlling interactions between microwave energy and the acid solution containing the sample.

Research has been conducted to identify these fundamental relationships and to develop methods that allow the analyst to predict, before programming and running the equipment, the conditions that will be generated during microwave digestion. This has been accomplished by measuring many of the parameters required to calculate the microwave power absorption by the mineral acids. Once the amount of energy that will be absorbed by a quantity of acid is determined, the equation is solved for the final temperature that will be reached by the sample at specific power settings. This method of predicting the temperature, or the time it takes to reach a particular temperature is useful in estimating the decomposition conditions [2].

This work has led to many new applications. Because microwave digestions occur in a well-defined, precisely controlled system, it is suitable for integration into automated applications. Acid digestion conditions have previously been too arbitrary for automation. With direct control of the power, the acid temperature, and the time for digestion, the microwave technique has become sufficiently structured so that it is possible to automate sample decomposition prior to instrumental analysis. New microwave-transparent vessels made of PFA Teflon and specifically engineered for this purpose permit the use of high temperatures (180–250 °C) and pressures (1000 kPa or 10 atm).

Because microwave energy is transferred directly to the acid, the reproducibility of decomposition conditions is better than can be achieved by traditional hotplate heating. Figure 1 shows the excellent reproducibility of sample conditions; it compares the temperature profile of two sets of six rice flour samples digested separately. The maximum difference between the sample temperatures at any point on the curve is 1.7%. Not only do the samples reach the same end point, but they achieve the same conditions at every point within this uncertainty.

Specific temperatures were identified for the rapid decomposition of the three basic components of biological and botanical matrices in nitric acid. Carbohydrate matrices decompose rapidly at a temperature of 140 °C, protein molecules rapidly decompose at 150 °C, and lipid molecules decompose at approximately 160 °C. These temperatures were determined by observing the nitric acid decomposition of each of these biological components separately. Three carbohydrates were used (soluble starch, amylopectin-amylose and glucose). The proteins were modeled by using Albumin (SRM 926, a pure protein), and tristearin (C-18, fatty acid ester) was used for the lipids. The oxidation by nitric acid was determined by measuring a rapid rise in pressure inside the closed container during a small change in solution temperature.

Biological materials decompose rapidly at temperatures that are closely related to their major components. Because the biological matrix is converted to CO_2_ and nitric acid is converted to NO_2_, these gaseous decomposition products produce a sharp rise in pressure with barely perceptible temperature changes. This rise is a good indication of the occurrence of decomposition. Figures 2 and 3 show the increase in pressure with the increase in temperature for bovine liver compared to albumin and to tristearin. The decomposition of bovine liver resembles tristearin rather than albumin. Tristearin requires a higher temperature to oxidize in nitric acid than does albumin. It appears that the more acid-resistant component of the matrix may be left intact if the conditions are not vigorous enough. Even at conditions that decompose the carbohydrate, protein and fatty acid molecules, certain organic moieties, such as the nitrobenzoic acids, remain after treatment with nitric acid. The nitrobenzoic acids are formed by the nitration of the aromatic rings of certain amino acids and they do not degrade further [3].

An additional test to observe the completeness of the digestion with time was carried out by decomposing albumin. It was first digested for 10 minutes under normal conditions, then cooled to room temperature, and digested a second time. Figure 4 shows that the pressure versus temperature curves for the two tests are very different. The second decomposition curve resembles the partial pressure of pure nitric acid heated under these same conditions; this comparison is shown in figure 5. The fact that no additional pressure was developed during the second heating indicates that all the oxidation occurred in the first 10 minute exposure to nitric acid under these specific conditions of temperature and pressure. These results indicate that rapid oxidation of biological samples can be achieved in approximately 10 minutes. The high temperature required for rapid oxidation of samples can be achieved reproducibly in a short period of time. These conditions require inert, closed vessels that can withstand substantial pressures and temperatures.

Because of the precise reproducibility of the conditions and decomposition, microwave dissolution has great potential as a tool in analytical laboratories. This new technique effectively addresses the problems of precision, accuracy and efficiency.

## Figures and Tables

**Figure 1:**
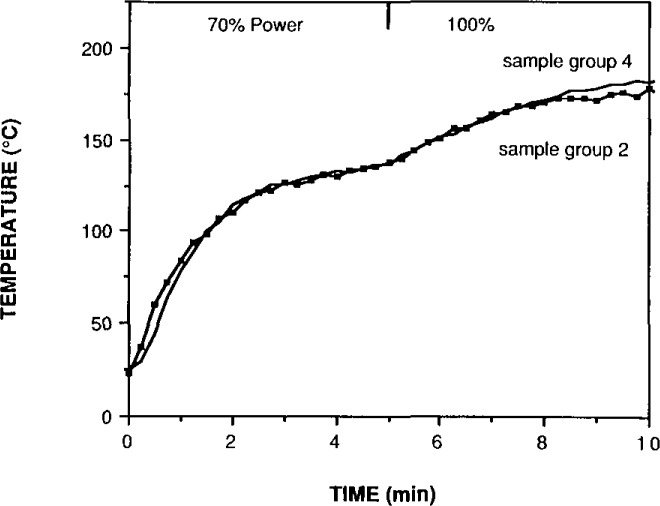
Temperature reproducibility of two sets of six samples each of 1 g of rice flour in 14 g of nitric acid.

**Figure 2:**
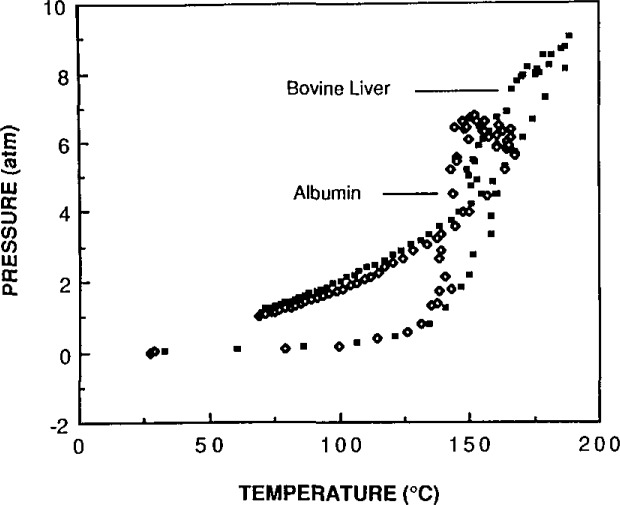
A comparison of the temperature versus pressure profile of acid decomposition of a biological tissue with that of a pure protein in the same acid.

**Figure 3:**
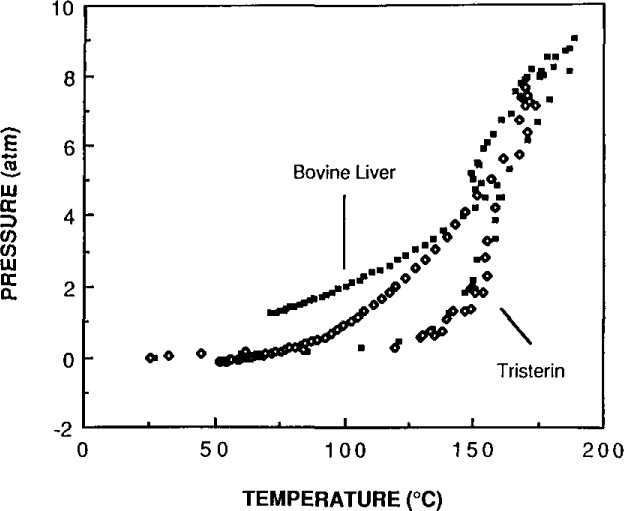
A comparison of the temperature versus pressure profile of acid decomposition of a biological tissue with that of a pure lipid.

**Figure 4:**
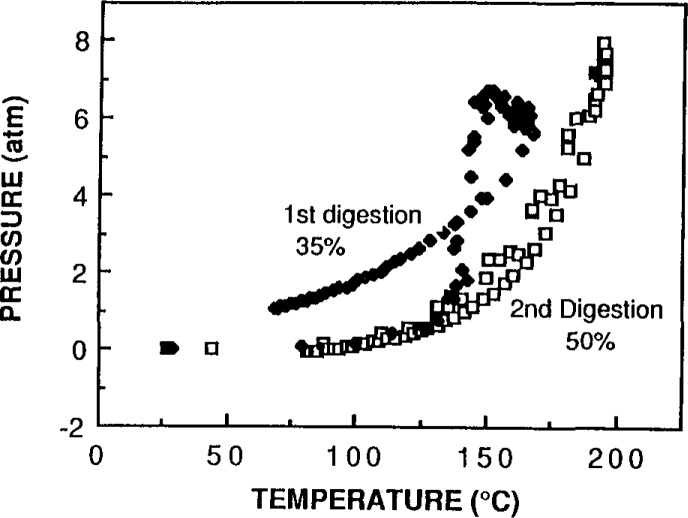
Temperature versus pressure profiles of the first and second digestions of a pure protein in nitric acid.

**Figure 5:**
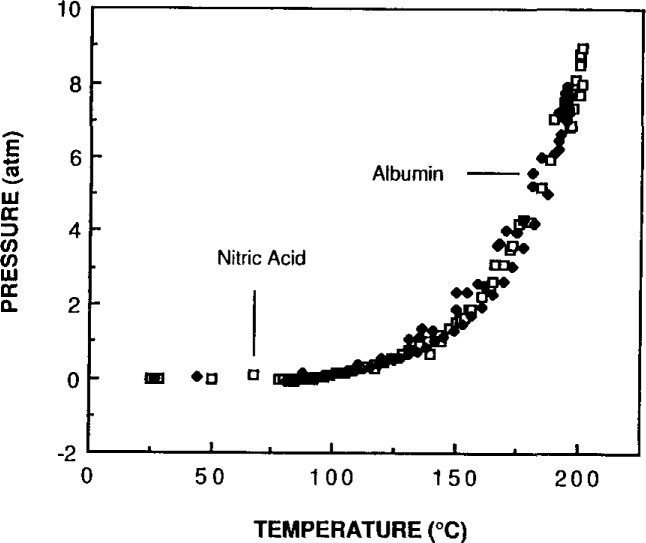
A comparison of the temperature versus pressure profile of the second digestion of a pure protein in nitric acid with that of nitric acid itself.
